# Odor habituation can modulate very early olfactory event-related potential

**DOI:** 10.1038/s41598-020-75263-7

**Published:** 2020-10-22

**Authors:** Kwangsu Kim, Jisub Bae, Youngsun Jin, Cheil Moon

**Affiliations:** 1 Department of Brain and Cognitive Sciences, Graduate School, Daegu Gyeungbuk Institute of Science and Technology (DGIST), 333, Techno Jung-Ang Daero, Hyeonpung-Myeon, Dalseong-Gun, Daegu, 711-873 South Korea; 2 Department of Psychology College of Social Sciences, Kyungpook National University, Daegu, South Korea; 3 Convergence Research Advanced Centre for Olfaction, Daegu Gyeongbuk Institute of Science and Technology (DGIST), Daegu, South Korea

**Keywords:** Neuroscience, Physiology

## Abstract

Odor habituation is a phenomenon that after repeated exposure to an odor, is characterized by decreased responses to it. The central nervous system is involved in odor habituation. To study odor habituation in humans, measurement of event-related potentials (ERPs) has been widely used in the olfactory system and other sensory systems, because of their high temporal resolution. Most previous odor habituation studies have measured the olfactory ERPs of (200–800) ms. However, several studies have shown that the odor signal is processed in the central nervous system earlier than at 200 ms. For these reasons, we studied whether when odors were habituated, olfactory ERP within 200 ms of odors could change. To this end, we performed an odor habituation behavior test and electroencephalogram experiments. In the behavior test, under habituation conditions, odor intensity was significantly decreased. We found significant differences in the negative and positive potentials within 200 ms across the conditions, which correlated significantly with the results of the behavior test. We also observed that ERP latency depended on the conditions. Our study suggests that odor habituation can involve the olfactory ERP of odors within 200 ms in the brain.

## Introduction

We are surrounded by a flood of odors from the environment in which we live. In this flood of odors, discerning the useful, vital odors from less useful, unimportant odors is essential for survival. Odor habituation is characterized by decreasing responses to odors to which the subject was continuously or repeatededly exposed. This phenomenon has important roles in odor perception. For example, odor habituation allows the endurance of malodorous environments, and ready distinguishing of new odors from background odors^1–3^.

To understand the processes underlying odor habituation, studying neuronal activities in the brain could be important. While odor habituation can be affected by top-down modulation, such as attention^4^, during habituation, neuronal activities in the central nervous system are changed earlier than those in the peripheral nervous system^3,5^. In the olfactory system, odorants are detected by olfactory sensory neurons (OSNs) in the olfactory epithelium. The detected signals are encoded in the olfactory bulb, and are finally processed in other areas of the brain. The piriform cortex (PC), entorhinal cortex, and orbitofrontal cortex (OFC) are known as major areas that perform olfactory processing in the brain, and are mainly located near deep brain areas^6–9^. Several studies have shown where brain activities change in odor habituation. In previous mouse and monkey studies, decreasing electrical and blood-oxygen-level-dependent (BOLD) signals in the PC were observed during odor habituation^1,10–14^. Furthermore, these studies showed that modulating neurons in the PC that express glutamate receptors affect odor habituation behavior^11,12,14^. Under odor habituation conditions in humans, BOLD signals decrease in the PC, and increase in the OFC^3,15^.

In addition to the brain areas involved in odor habituation, temporal changes in the brain signals during odor habituation could be important. Neural activities in brain areas involved in the olfactory process change with time^16,17^. In mice, the electrical signal of odors observed in the brain within 200 ms increases or decreases during odor habituation^12,18–21^. These studies provide evidence that brain signals of odors within 200 ms can be related to odor habituation. In humans, the measurement of event-related potentials (ERP) has been widely used to understand sensory habituation, because ERP data have high temporal resolution, and directly reflect electrical neuronal signals. An examination of ERP data could provide evidence of temporal changes in brain signals during odor habituation. In recent studies, ERP signals with negative potential at (200–700) ms (N1) and positive potential at (300–800) ms (P2) changed during odor habituation^22,23^. These studies in rodents and humans suggest that during odor habituation processing, temporal changes in the brain signals can exist. However, the studies also imply that different temporal windows exist for rodents and humans, respectively.

Some lines of evidence suggest that similar to animal studies, the time of odor signal processing in the human brain could be earlier than 200 ms. Previous odor habituation ERP studies in humans^22–25^ mainly focused on odor signals in the brain after 200 ms, because they were partially based on previous studies of OSNs from the olfactory epithelium^26,27^. These studies observed that the time of odor signal processing by OSNs was about 300 ms. However, they did not consider factors affecting processing time, such as airflow, mucus, and odorant receptor family. Moreover, there was a discrepancy between the time ranges of ERP signals in these studies, and the time of odor signal processing in the primary (e.g. PC) and secondary olfactory cortex (e.g. OFC). Recent studies based on electroencephalograms (EEG) and Magnetoencephalography (MEG) showed that the olfactory signal could reach the PC in about 40 ms^16,17^. Furthermore, direct neuronal electrical signal data from human epileptic brains suggested that the PC and OFC process odor information earlier than in 200 ms^28^. Behavioral evidence indicates as well that respiration can be controlled in response to odors within 160 ms, and odor discrimination could happen within 400 ms^29^. These lines of evidence show that the odor signal is processed in the brain earlier than in 200 ms, and suggest the existence of an early ERP signal in the brain related to odor habituation.

Therefore, our purpose in this study was to find whether the olfactory ERP within 200 ms of odors could change when the odors were habituated. If the odor signal process of the brain from 40 ms was involved in odor habituation, the olfactory ERP within 200 ms of odors would change. Otherwise, the olfactory ERP of odors within 200 ms would not change. We conducted a behavior test and ERP measurements during odor habituation in human participants. Previous studies have shown that continuous exposure to the same odors during 30 s induce changes in the brain odor signal and behavioral responses^10,11^. Thus in our experimental scheme, odors were offered during the entire 30 s to induce odor habituation. Data on the behavioral responses and brain signal induced by the offered odors just after the 30 s were collected. The amplitude and latency of olfactory ERP were analyzed at (40–200) ms to examine changes in the brain signal during odor habituation. The maximum peaks of negative potential (NP) and positive potential (PP) at (40–200) ms were chosen because 40 ms is the earliest time point when the electrical signal induced by odors can be detected in the human brain, whereas 200 ms is the time point when behavioral responses may already have occurred, depending on the odor intensity and signal in the olfactory cortex^16,17,29^. We also examined the correlation between the results of the behavior test and ERP data within 200 ms, to check that these ERP data were actually related to the behavior test.

## Methods

### Participants

Fourteen participants (six men, eight women, aged between 19 and 29 years, mean age 21.1, SD 2.4) were recruited from the Daegu Gyeongbuk Institute of Science and Technology. Among them, 13 participants were included in this study, because the data of one male participant was excluded because of technical problems. All participants were right-handed, and had normal olfactory function, as determined by the odor threshold and odor discrimination tests in the Sniffin Sticks test. Participants provided written informed consent in advance. This study was approved by the Daegu Gyeongbuk Institute of Science and Technology (DGIST-180524-h-005-03) confirming that all methods were performed following relevant guidelines and regulations in advance^30^.

### Odor preparation and delivery

**Figure 1:**
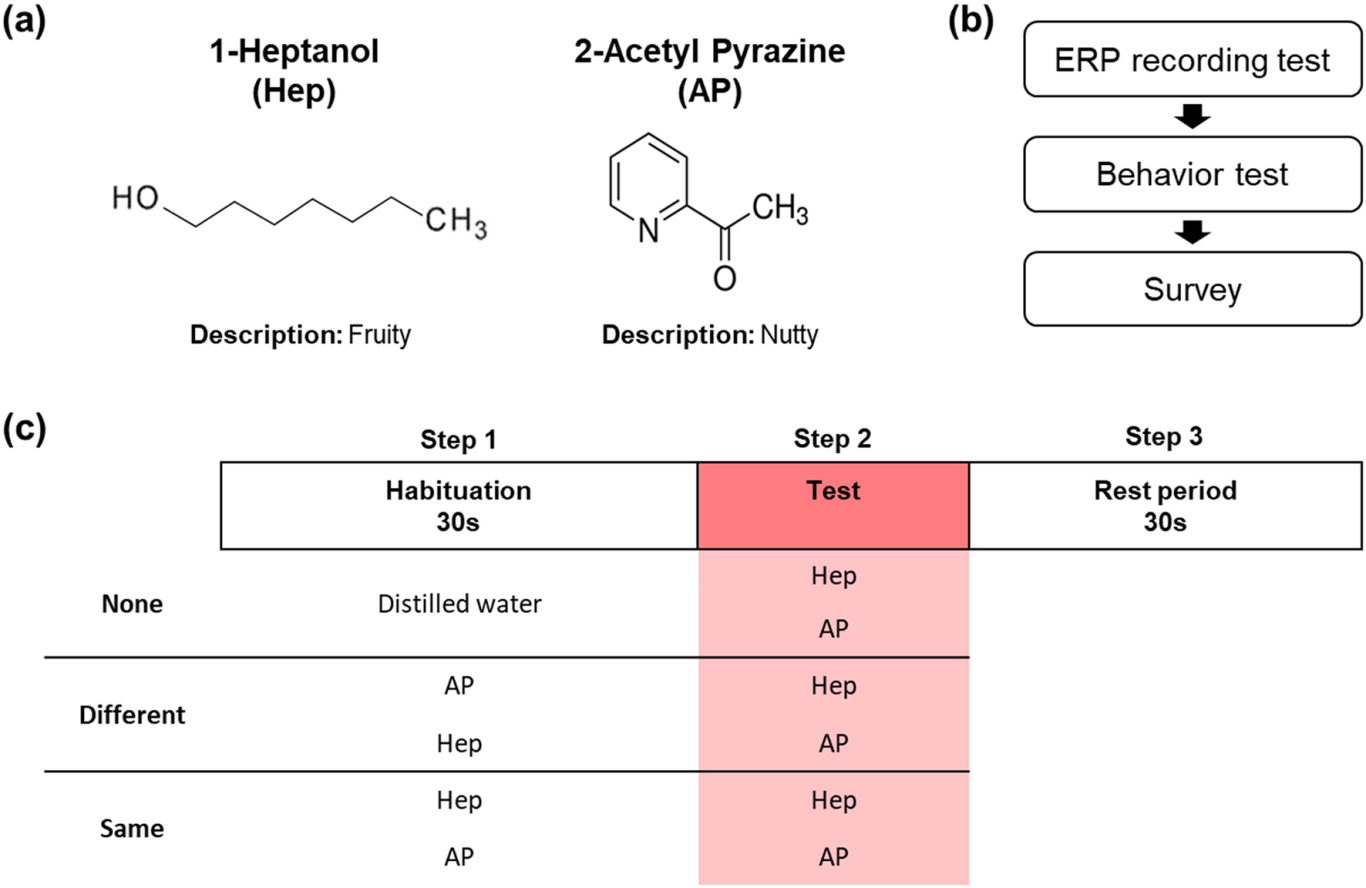
Experimental scheme. (**a**) 1-heptanol and 2-acetyl pyrazine used in this experiment. These odorant compounds have different chemical structures and descriptions (The Good Scents Company Information System). (**b**) Overview of tests used in this study. (**c**) The ERP recording test and behavior test consisted of three steps. The first step was habituation. To induce habituation, odors or distilled water were offered continuously for 30 s. The second step was the test. One of the two odors was offered to measure the intensity and brain signal. The last step was 30 s of rest period, before the next round of the experiment. There were three different habituation conditions. “None”: distilled water was offered in the first step, and one of the two odors in the second step. “Different”: if 2-acetyl pyrazine was offered in the first step, 1-heptanol was offered in the second step, and vice versa. “Same”: the same odorant compound was offered in the first and second steps.

This study used 1-heptanol (Sigma, CAS 111-70-6, Lot #STBD9537V) and 2-acetylpyrazine (Sigma, CAS 22047-25-2, Lot #MKCB169V) (Fig. 1a). We chose these structurally and perceptually different odors to avoid cross adaptation. 1-heptanol was diluted in mineral oil (Sigma, Lot# MKBZ6778V) to 10% (v/v) to be aliquoted in 1.5 mL tubes and 20% concentration for use in a custom-built olfactometer. The 2-acetylpyrazine was diluted to 0.1% (w/v) in distilled water for both purposes. In ERP recording test, in the habituation step, odors were offered as a tube by participants’ hand. In the test step, odors were offered by the olfactometer. The olfactometer was linked to the mask (without canula), which participants wore. All odors were delivered to the participant just after an inhalation peak and until the next inhalation peak (approximately 2 s). In behavior test, in both the habituation and the test steps, odors were offered to participants by an experimenter (Fig. 1). When odors were offered by the olfactometer, airflow speed was 5.18 m/s.

### Experimental procedure

All tests were conducted in a ventilated and soundproof chamber. Participants were instructed to maintain their natural respiration during the tests. They participated in an ERP recording test, behavior test, and survey (Fig. 1b). The ERP recording and behavior test each consisted of three steps: habituation, test, and rest. In the habituation step, 1-heptanol, 2-acetyl pyrazine, or distilled water was offered to participants continuously during the entire 30 s. Then in the test step, one of the two odors was offered to measure the odor intensity and brain signal, considering participants’ respiration. In the rest step, no odors or distilled water were offered during the entire 30 s before the next trial (Fig. 1c).

Based on which odor was offered in the habituation and test steps, there were three conditions. If distilled water was offered in the habituation step and one of the two odors was offered in the test step, the condition was called “None”. If different odors were offered in the habituation and test steps, the condition was defined as “Different”. Lastly, if the same odor was offered in the habituation and test steps, the condition was called “Same” (Fig. 1c). The “None” condition was for checking the ERP of odors without any pre-existing stimulus. Therefore, the signal-to-noise ratio (SNR) of ERP induced by odors was calculated in the “None” condition. “Different” condition was designed to examine whether the offering of odors itself could affect odor habituation and ERP of odors. The “same” condition was to examine the ERP of odors with the same pre-existing stimulus.

In the ERP recording test, participants were sitting on a chair in the chamber, and wearing a mask that was linked to the olfactometer. The participant’s respiration was monitored by a respiration belt. Participants were instructed to hold a tube, which contained one of the odors or distilled water. In the habituation step, participants were instructed to smell the odor from the tube during the entire 30 s; they were guided by visual cues on a monitor in front of them: when the visual cue disappeared, the participants were instructed to stop smelling the odor, and to immediately close their eyes. In the test step, odors were offered by the olfactometer. The stimulus was delivered to the participant just after an inhalation peak, and until the next inhalation peak (approximately 2 s). EEG was recorded during the test step. We used the olfactometer only in the test step. This was because the purpose of our study was to compare the ERP of odors when the odors were habituated. Two sessions under each of the three different conditions were conducted for each participant. In a single session, 16 trials of ERP recording were conducted for each participant. Each session lasted approximately 16 min, and after three sessions, there was a short break. The order of the sessions was randomized to minimize sequence effects across participants, because only one odor was used in a session.

During the behavior test, participants sat on a chair in the chamber, and wore a blindfold, to prevent possible visual cues during odor exposure. An experimenter offered odors and distilled water. All stimuli were contained in 1.5 mL tubes, which were located at 3 cm from the participant’s nose when the stimulus was offered. 1-heptanol, 2-acetylpyrazine, or distilled water were offered during the entire 30 s in the habituation step. Then, one of the odors was offered to the participant in the test step just after an inhalation peak, and until the next inhalation peak (approximately 2 s). Participants were instructed to mark the intensity of the odor in the test step on a 9-point Likert scale questionnaire. Each of the three conditions was tested once, and the order of conditions was randomized across participants.

In the survey, the participants were asked to evaluate the pleasantness and intensity of the 1-heptanol and 2-acetylpyrazine odors on a 9-point Likert scale questionnaire.

### Electroencephalogram recording

The odor stimulation in the test step was recorded by EEG. EEG signals were digitized via an EEG amplifier (ActiveTwo, BioSemi, Amsterdam, the Netherlands). EEG signals were recorded with Ag/AgCl scalp electrodes from 64 positions of the international 10/20 system (Fp1, AF7, AF3, F1, F3, F5, F7, FT7, FC5, FC3, FC1, C1, C3, C5, T7 (T3), TP7, CP5, CP3, CP1, P1, P3, P5, P7, P9, PO7, PO3, O1, Iz (inion), Oz, POz, Pz, CPz, Fpz, Fp2, AF8, AF4, Afz, Fz, F2, F4, F6, F8, FT8, FC6, FC4, FC2, FCz, Cz, C2, C4, C6, T8 (T4), TP8, CP6, CP4, CP2, P2, P4, P6, P8, P10, PO8, PO4, and O2) on a BioSemi headcap (64 ch, BioSemi). Eye blinks (electrooculographic signals) were measured at approximately 2 cm above the outer canthus of the right eye. The sampling rate was 2,048 Hz, and the signals were analog-filtered via a 0.15 high-pass filter and a 100 Hz low-pass filter. A conductive electrolyte gel was used for a stable connection between the scalp and the electrodes. The impedance of each electrode was below 10 kΩ. The electrophysiological activity was referenced to the common average of all channels.

### Electroencephalogram preprocessing

EEG data were downsampled from (2048 to 512) Hz. An offline bandpass filter was (1–20) Hz to minimize noises caused by muscle artifacts and skin potential. The bandpass filter was applied with a cut-off frequency (− 6 dB point) of 0.5–20.5 Hz after extracting epochs, and the type of the applied filter was FIR (finite impulse response) filter. The default order was used for the FIR filter in EEGLAB^31^. EEG data were segmented into epochs. Each epoch had a 500 ms pre-stimulus period, and a 1500 ms post-stimulus period. Starting stimulus points were set at the participant’s exhalation peaks in every trial, because an exhalation peak was the turning point to restart inhalation. Before averaging the epochs of the respective conditions, filtered EEG data contaminated with eye blinks (> 40 μV), or mismatched between the respiration cycle and odor offering time, were discarded. Data from (– 500 to 0) ms in each epoch were used for baseline correction.

### Event-related potentials

After EEG preprocessing, over 23 trials of EEG data per condition were averaged. Therefore, each participant had three ERP datasets under the three conditions (“None”, “Different”, and “Same”). NP and PP peaks were determined as the most negative and the most positive peaks, respectively, between (40 and 200) ms in the ERP data of each participant according to previous studies^16,17,29^. For the comparison with negative and positive peaks in the previous odor habituation study^23^, the most negative peak between (200 and 700) ms (N1) and the most positive peak between (300 and 800) ms (P2) were also selected.

### Statistics

The results are presented as mean ± sem. The significance in statics was marked as * for p < 0.05, ** for p < 0.01, and *** for p < 0.001. The odor intensity in the behavior test was analyzed by one-way repeated measure ANOVA (RMANOVA), to find the significantly different intensity of conditions. The intensity scores of “None,” “Different,” and “Same” condition in the behavior test were the factors of the RM ANOVA. Bonferroni’s correction was used in the RM ANOVA of the intensity scores across the conditions. Bonferroni’s test was conducted as a post hoc test, and the level of correction for the Bonferroni correction was 0.017(0.05/3). In addition, power analysis of the sample size was conducted for the behavior test. One sample *t* test was used for the SNR of ERP to compare noise level (value 1). The ERP amplitude and latency across conditions were analyzed by RMANOVA, to find significantly different ERP amplitudes and latency of channels across the conditions. The NP peaks of “None,” “Different,” and “Same” condition in between (40 and 200) ms were the factors of the RM ANOVA in each channel. Likewise, PP peak, NP latency and PP latency of the conditions were the factors of the RMANOVA in each channel. The Greenhouse–Geisser correction was applied when there was nonsphericity in the RMANOVA. Original degrees of freedom, F values, uncorrected p values, Greenhouse–Geisser ε values, and partial eta-squared effect sizes (pη^2^) were reported. Bonferroni’s correction was used in the RM ANOVA of the ERP analysis in each channel. Bonferroni’s test was conducted as a post hoc test, and the level of correction for the Bonferroni correction was 0.017 (0.05/3). The intensity score of the behavior test and the ERP amplitude or latency across conditions were compared by Pearson correlation analysis. The intensity and pleasantness of the two odors were analyzed by a two-tailed *t* test.

### Software

Electrophysiological data were analyzed using MATLAB 2016b, in conjunction with toolboxes that included EEGLAB^31^. MATLAB was also used for statistical analysis.

## Results

### The perceived intensity of the odor decreased when the same odor was offered again

**Figure 2:**
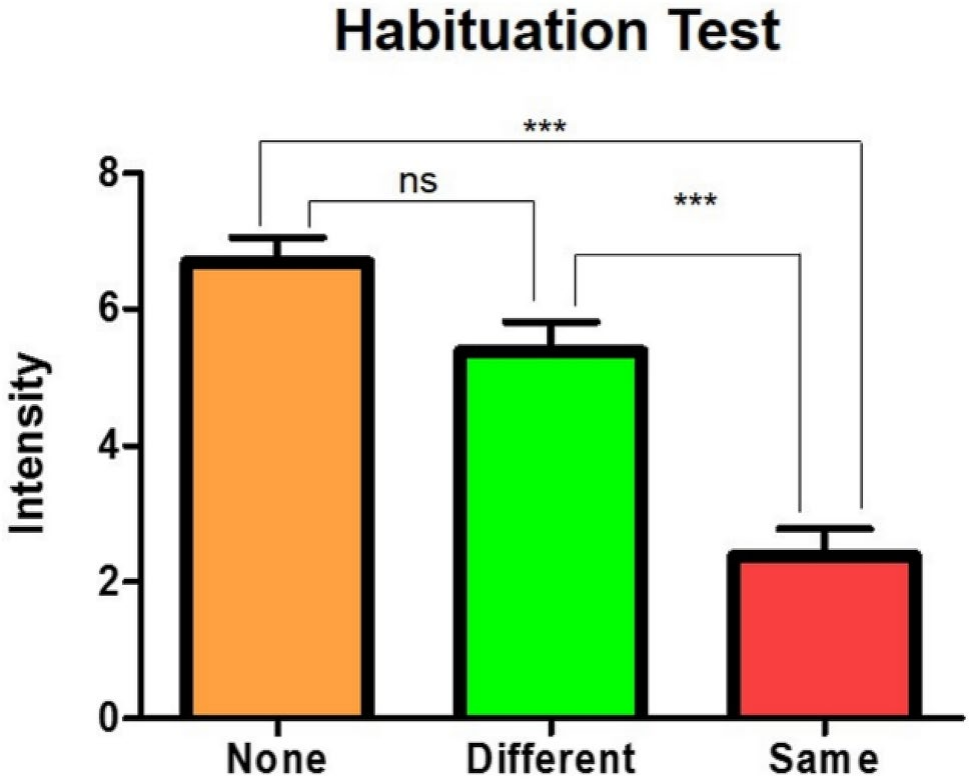
Decrease in odor intensity when the same odor was offered. The intensities of the odors offered in the test step were compared across the three conditions. Odor intensity was significantly lower under the “Same” condition, than under the “None” and “Different” conditions, but did not differ significantly between the “None” and “Different” conditions. ***p < 0.001.

To verify whether odor habituation occurred under the “Same” condition, the perceived intensities of the odors offered in the behavior test were compared across the conditions (Fig. 2). Intensity scores were significantly different across the conditions (F[2|38] = 37.56, p < 0.0001, ε = 0.81, pη^2^ = 0.76; RMANOVA). The power value for 13 sample sizes in the behavior test was 1 (significance level 0.05). The intensity score was significantly lower in the “Same” condition, than in the “None” condition (T = 8.13, p < 0.0001; Bonferroni’s test) and “Different” condition (T = 5.70, p < 0.0001; Bonferroni’s test). The pleasantness (T[12] = 0.71, p = 0.49) and intensity (T[12] = 0.28, p = 0.78) scores of 1-heptanol were not significantly different from those of 2-acetyl pyrazine [Fig. S1 of the Supplementary Information (SI)].

### Significant amplitude and latency changes of negative and positive potentials within 200 ms

**Figure 3:**
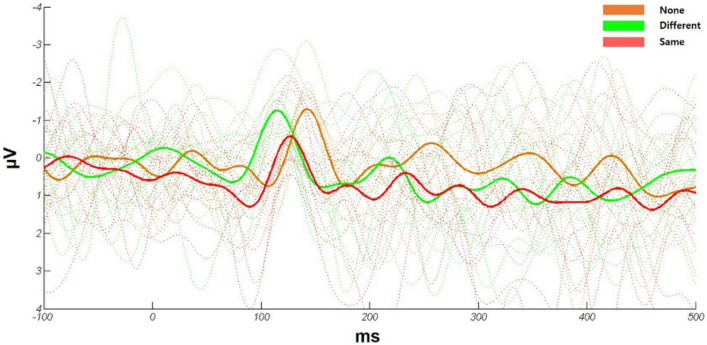
Grand average event-related potential (ERP) across all participants (n = 13) under the “None”, “Different”, and “Same” conditions. Dotted lines show the ERPs of individual participants. Thick lines are the means of all participants under the three conditions. Mean ERP of odors under each condition data from the C6 channel is shown.

To examined the olfactory ERP changes within 200 ms of the odor when the odor was habituated, ERP amplitudes and latencies were compared across the three conditions (“None”, “Different”, and “Same”) in a total of 64 electrodes. In the grand average, each participant’s ERPs were fitted based on negative potential peaks. The time point of NPs in the grand average was also fitted based on the mean latency of all participants’ negative potentials in each condition (Fig. 3). To characterize ERP signals within 200 ms, we calculated the SNR of peaks of the NP or PP from (40–200) ms. We found that the SNR of the NP (1.368 ± 0.0366) and PP (1.343 ± 0.0367) was statistically higher than the noise level (value 1) (Table 1).

**Table 1:** Significant higher value in SNR of ERP (40–200) ms.

SNR of ERP (40–200) ms							
Negative Potential				Positive Potential			
Mean	Df	SEM	P value	Mean	Df	SEM	P value
1.368	12	± 0.0366	< 0.0001	1.343	12	± 0.0367	< 0.0001

In toto, 64 channels were used. SNR of NP and PP were significantly higher than the noise level (value 1) in the one sample *t* test from (40–200) ms across all participants.

We first examined differences in NP among the conditions. We found that four channels showed significant differences (Table 2a). Specifically, three channels showed significantly different amplitudes across the conditions: C2, C6, and F5 channels (Table 2a, left panel; C2 channel: F[2|38] = 3.97, pη^2^ = 0.25; C6 channel: F[2|38] = 3.49, pη^2^ = 0.23; F5 channel: F[2|38] = 3.79, pη^2^ = 0.24; p < 0.05, ε > 0.75 each, RMANOVA). In C2 and C6, the NP amplitude tended to be lower under the “Same” condition, than under the other two conditions. In the C2 channel, the NP amplitude was significantly lower under the “Same” condition, than under the “None” condition (T = 2.81, p < 0.05; Bonferroni’s test). On the other hand, in the F5 channel, the NP amplitude tended to be lower under the “None”, than under the other two conditions. The CP6 channel showed significantly different latencies across the conditions (Table 2a, right panel; F[2|38] = 3.76, p = 0.038, ε = 0.72, pη^2^ = 0.24; RMANOVA). In the CP6 channel, the NP latency under the “None” condition was significantly later than the latency under the “Different” condition (T = 2.72, p < 0.05; Bonferroni’s test), and tended to be later under the “None” condition, than under the “Same” condition.

**Table 2:** Channels with significant differences across the conditions in the amplitude and latency of NP and PP for (40–200) ms.

ERP component	Channel	Amplitude (µV)				Latency (ms)			
		None	Different	Same	F-value	None	Different	Same	F-value
**(a)**									
**Negative potential (40–200) ms**	Right hemisphere								
	C2	− 1.38	− 0.93	− 0.59	3.97*	131	125	100	1.11 (ns)
	C6	− 1.29	− 1.26	− 0.57	3.49*	143	114	126	1.36 (ns)
	CP6	− 1.41	− 1.25	− 1.18	0.62 (ns)	146	100	128	3.76*
	Left hemisphere								
	F5	–1.51	–2.68	–2.60	3.79*	97	106	125	0.87 (ns)
**(b)**									
**Positive potential (40–200) ms**	Right hemisphere								
	AF8	2.80	2.65	2.46	0.14 (ns)	102	156	116	3.73*
	C4	1.47	1.47	2.06	3.65*	105	121	130	0.57 (ns)
	CP6	1.11	1.38	1.94	4.62*	119	128	120	0.09 (ns)
	Left hemisphere								
	FC5	1.71	1.12	0.85	3.71*	136	117	119	0.41 (ns)
	C3	0.98	1.27	1.18	0.47 (ns)	134	87	141	3.71*
	C5	1.12	1.02	0.79	1.21 (ns)	148	112	100	3.50*
	CP3	1.43	1.78	1.02	3.95*	131	118	152	1.32 (ns)
	CP1	1.20	1.96	1.45	5.59*	97	102	136	2.21 (ns)
	P1	1.23	2.45	1.90	6.16**	110	109	108	0.01 (ns)
	P3	1.77	2.46	1.93	3.52*	128	132	130	0.02 (ns)
	Central position								
	Cz	1.91	2.30	1.90	0.57 (ns)	124	106	152	4.27*
	Pz	1.43	2.87	2.02	6.29**	111	112	128	0.47 (ns)
	CPz	1.56	2.34	1.84	2.59 (ns)	107	108	160	7.68**

In toto, 64 channels were used. a. NP. Four channels showed significant differences in NP amplitudes or latency. C2 and C6 showed significantly higher NP amplitude under the “None” or “Different” condition, than under the “Same” condition in the right hemisphere. F5 showed significantly higher NP amplitude under “Different” or “Same”, than under the “None” condition in the left hemisphere. CP6 showed significantly different NP latency across conditions in the right hemisphere. b. PP. Thirteen channels showed significant differences in PP amplitudes or latency. C4 and CP6 showed significantly higher PP amplitude under the “Same” condition, than under the other two conditions in the right hemisphere. FC5, CP3, CP1, P1, and P3 showed significantly different among conditions in the left hemisphere. FC5 and CP3 showed higher values under “None” and “Different” conditions, than under the “Same” condition; CP1, P1, and P3 showed higher values under “Different” condition, than under the other two conditions. Pz in the central position showed a lower value under the “None” condition, than under the other two conditions. AF8, CP6, C3, C5, Cz, and CPz showed significant differences in latency. AF8 and CP6 showed higher values under “Different” condition, than under the other two conditions in the right hemisphere. C3 showed a lower value under “Different” condition, whereas C5 showed a higher value under the “None” condition, in comparison with the other two conditions in the left hemisphere. Cz showed a lower value under “Different” condition, while CPz showed a higher value under the “Same” condition, in comparison with the other two conditions.

We also examined differences in PP among the conditions. Thirteen channels showed significant differences (Table 2b). Specifically, eight channels showed significantly different PP amplitudes across the conditions: C4, CP6, FC5, CP3, CP1, P1, P3, and Pz channels (Table 2b, left panel; C4: F[2|38] = 3.65, pη^2^ = 0.23; CP6: F[2|38] = 4.58, pη^2^ = 0.28; FC5: F[2|38] = 3.71, pη^2^ = 0.24; CP3: F[2|38] = 3.95, pη^2^ = 0.25; CP1: F[2|38] = 5.59, pη^2^ = 0.32; P1: F[2|38] = 6.16, pη^2^ = 0.34; P3: F[2|38] = 3.52, pη^2^ = 0.23; Pz: F[2|38] = 6.29, pη^2^ = 0.34; p < 0.05, ε > 0.75 each, RMANOVA). Among these eight channels, FC5 and CP3 showed a tendency for the PP amplitude to be lower under the “Same” condition than under the other two conditions. Bonferroni’s post-test also suggested that FC5 (T = 2.66, p < 0.05) and CP3 (T = 2.81, p < 0.05) showed significant differences in the amplitudes of the “None” and “Same” conditions. The C4 and CP6 channels showed a tendency for the PP amplitude to be higher under the “Same” condition, than under the other two conditions. Bonferroni’s post-test also suggested that CP6 (T = 2.97, p < 0.05) showed significant differences in the amplitudes of the “None” and “Same” conditions. The remaining channels showed a different pattern: they had the highest amplitude under the “Different” condition. In the CP1, P1, and Pz channels, the PP amplitude was significantly higher under the “Different” condition, than the “None” condition (CP1: T = 3.28, p < 0.01; P1: T = 3.50, p < 0.01; Pz: T = 3.53, p < 0.01; Bonferroni’s test). Five channels had significant differences in PP latency across conditions: AF8, C3, C5, Cz, and CPz channels (Table 2b, right panel; AF8: F[2|38] = 3.74, pη^2^ = 0.24; C3: F[2|38] = 3.71, pη^2^ = 0.24; C5: F[2|38] = 3.50, pη^2^ = 0.23; Cz: F[2|38] = 4.27, pη^2^ = 0.26; CPz: F[2|38] = 7.69, pη^2^ = 0.39; p < 0.05, ε > 0.75 each, RMANOVA). In C3, Cz, and CPz, latency tended to be slower under the “Same” condition, than under the other two conditions. In the CPz channel, the PP latency was significantly slower under the “Same” condition, than under the “Different” condition (T = 3.33, p < 0.01; Bonferroni’s test) and “None” condition (T = 3.46, p < 0.01; Bonferroni’s test). In the AF8 channel, the PP latency was slower under the “Different” condition, than under the other two conditions; in the C5 channel, the PP latency was slower under the “None” condition, than under the other two conditions. In post-test, the PP latency in the AF8 channel was significantly slower under the “Different” condition, than under the “None” condition (T = 2.64, p < 0.05; Bonferroni’s test).

These results suggest that during odor habituation, the NP and PP within 200 ms changed. In the case of NP, the amplitude under the “Same” condition changed in two channels (C2 and C6), compared to that under the other two conditions. In the case of PP, the amplitude under the “Same” condition changed in four channels (FC5, CP3, C4, and CP6), while the latency under the “Same” condition changed in three channels (C3, Cz, and CPz).

### Changed NP and PP patterns across the conditions are related to the behavior test

Although our analysis showed that during odor habituation, NP and PP within 200 ms changed, further observations and comparison between ERP (i.e., NP, PP) and the behavior test were necessary to understand their relationships. Using correlation analysis, we analyzed the relation between the amplitude and latency of ERP across the conditions and behavior results.

**Figure 4:**
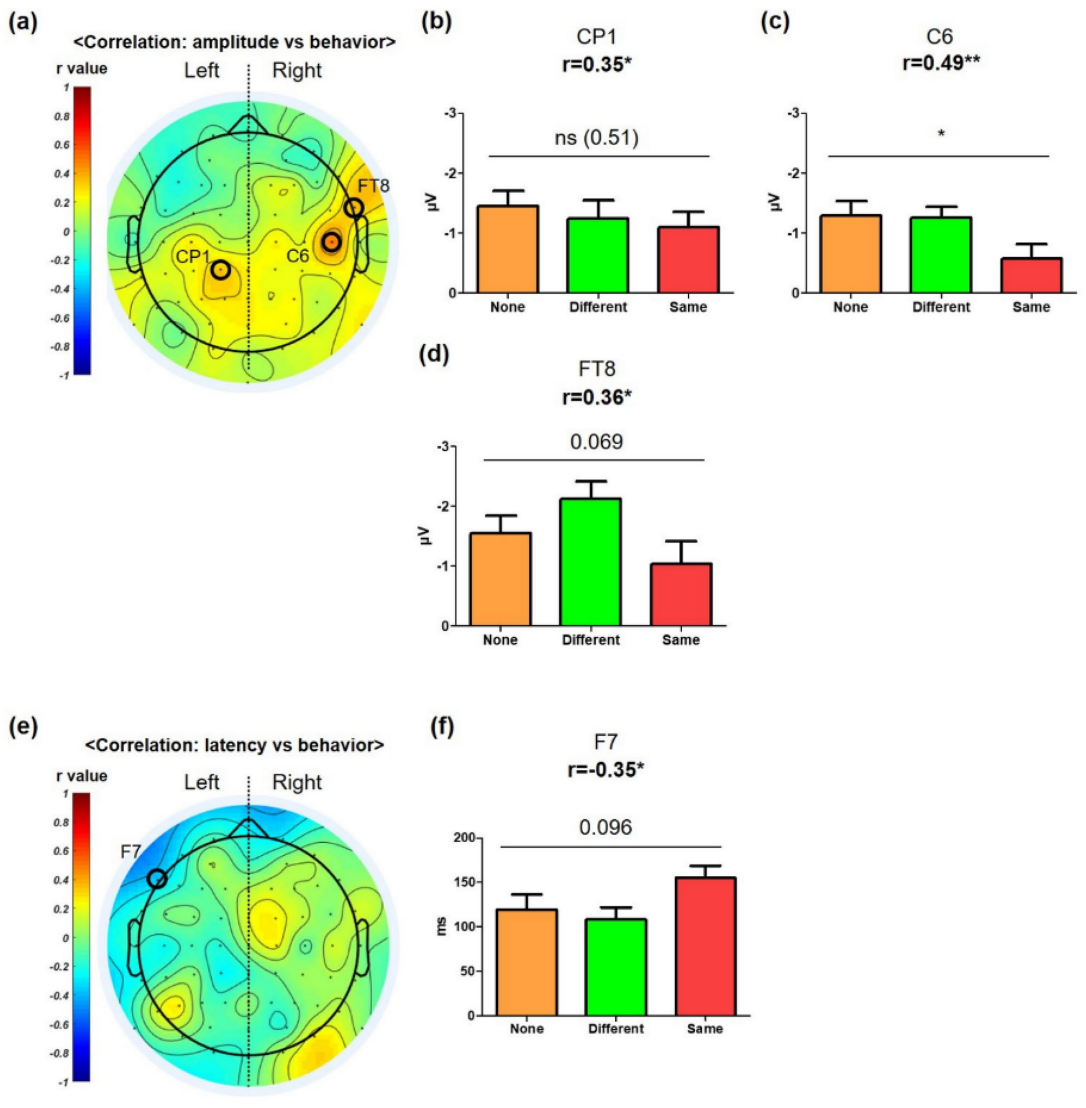
Correlation pattern between NP and behavior [(40–200) ms NP vs. Habituation Test]. Topographical patterns of correlation between NP and behavior. Encircled channels showed statistically significant correlations. (**a**) Topographical patterns of correlation between NP amplitude and the behavior. Three channels showed significant correlations (CP1, C6, and FT8). (**b**–**d**) CP1, C6, and FT8 significantly correlated with behavior (r value ≥ 0.35). In RMANOVA, C6 also showed a statistically significant decrease, and FT8 showed a tendency to decrease under the “Same” condition. (**e**) Topographical patterns of correlation between NP latency and behavior. f. F7 showed a significant negative correlation with behavior (r value =  − 0.35). F7 was not significantly different in NP latency across conditions. *p < 0.05, **p < 0.01.

In three channels, we found significant positive correlations of the NP amplitude with the behavior among the whole channesls (Fig. 4a–d; CP1: r = 0.35, p = 0.031; C6: r = 0.49, p = 0.002; FT8: r = 0.36, p = 0.026), and the C6 channel showed significantly different NP amplitudes across the conditions (Fig. 4c). Patterns of significant positive correlations of the NP amplitude with the behavior in the right hemisphere within 200 ms were maintained in patterns of the correlations of the NP amplitude with the behavior between (200 and 700) ms (Fig. 4a, and Fig. S2a of the SI). F7 channel showed a significant negative correlation between NP latency and the results of the behavior test, but there were no significant differences across the conditions (Fig. 4f).

**Figure 5:**
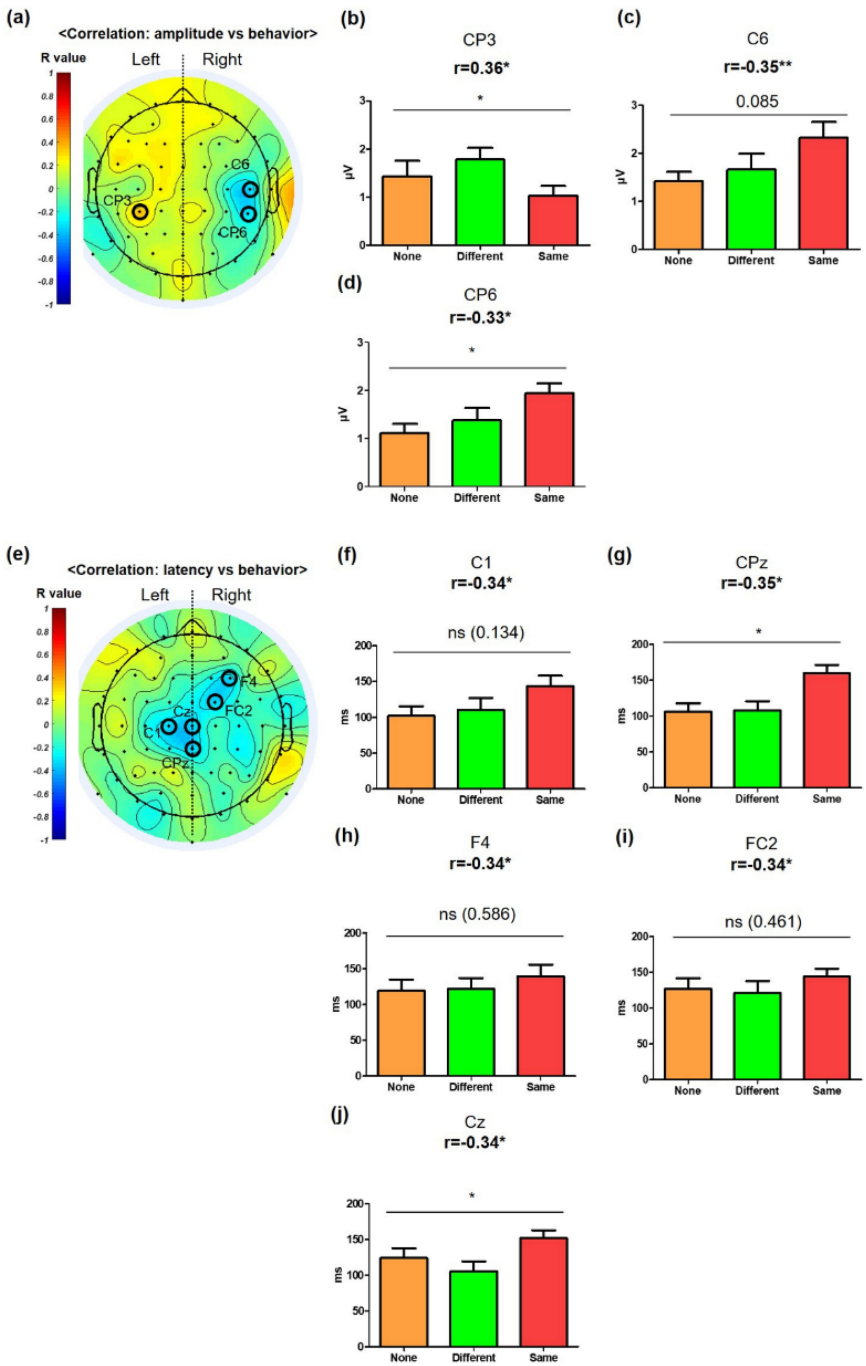
Correlation pattern between PP and behavior [(40–200) ms PP vs. Habituation Test]. Topographical patterns of correlation between PP and behavior. Encircled channels showed statistically significant correlation analysis. (**a**) Topographical patterns of correlation between PP amplitude and behavior. Three channels showed significant correlations (CP3, C6, and CP6). (**b**–**d**) CP3, C6, and CP6 significantly correlated with behavior (|*r* value| ≥ 0.33). In RMANOVA, CP3 showed a statistically significant decrease, and CP6 showed a statistically significant increase under the “Same” condition, whereas C6 showed a tendency for an increase under this condition. (**e**) Topographical patterns of correlation between PP latency and behavior. Five channels showed significant correlations with behavior (C1, CPz, F4, FC2, Cz). (**f**–**j**) C1, CPz, F4, FC2, and Cz significantly correlated with behavior (r value ≥ 0.34). In ANOVA, CPz showed a significant decrease, and Cz showed a significant increase under the “Same” condition. C1, F4, and FC2 showed a tendency for an increase under the “Same” condition. *p < 0.05, **p < 0.01.

In three channels, we found significant correlations of the PP amplitude with behavior among the whole channels (Fig. 5a): the CP3 channel showed positive correlation (Fig. 5b; r = 0.36, p = 0.023), whereas the C6 and CP6 channels showed negative correlation (Fig. 5c,d; C6: r =  − 0.34, p = 0.028; CP6: r =  − 0.33, p = 0.042). Moreover, the CP3 and CP6 channels showed significantly different PP amplitudes across the conditions, and that of the C6 channel had a similar tendency to that of the CP6 channel, although there was less statistical significance in the C6 channel. Patterns of significant positive and negative correlations of the PP amplitude with the behavior in the left and right hemispheres within 200 ms were maintained in the patterns of the correlations of the NP amplitude with the behavior between (300 and 800) ms (Fig. 5a, and Fig. S3a of the SI). Five channels showed a significant correlation between the PP latency and the results of the behavior test (Fig. 5e). Specifically, C1, CPz, F5, FC2, and Cz channels showed negative correlation (Fig. 5f–j, C1: r =  − 0.34, p = 0.036; CPz: r =  − 0.35, p = 0.028; F5: r =  − 0.34, p = 0.034; FC2: r =  − 0. 34, p = 0.032; Cz: r =  − 0.34, p = 0.032). In the CPz and Cz channels, we also found significantly different PP latency across the conditions (Fig. 5g,j).

## Discussion

We found that when the odors were habituated, the ERP signals of odors within 200 ms changed, and that these changes were related to odor habituation. For the behavior test, we chose 1-heptanol and 2-acetylpyrazine, which are perceptually and structurally different odors, and confirmed that the intensity of odor was significantly decreased under the “Same” condition, but not under the “Different” and “None” conditions (Fig. 2). This result suggests that odor habituation occurred mainly when the same odors were repeated, whereas cross adaptation caused by the two odors was barely detectable. This result is in line with previous odor habituation studies^3,32^. Under our experimental setting, we found that depending on the conditions, ERPs differed within 200 ms (Table 2). In several channels, the amplitude and latency of NP or PP were changed within 200 ms under the “Same” condition, compared with under the other conditions. To examine whether these ERP changes were related to the behavior, we performed a correlation analysis between the ERPs and behavior results. We found a significant correlation between the ERPs within 200 ms and the behavior results, mainly in channels located in the right temporal and parietal lobe areas (Figs. 4, 5), implying that the information of odor habituation may be processed centrally at early time points.

Our results suggest that odor habituation condition can modulate ERP signal in the brain at very early time points (within 200 ms). This evidence implies the involvement of the primary and secondary olfactory cortex. Previous studies regarded the involvement of the central nervous system as the major mechanism of odor habituation^3,11–14^. Not only can neuronal activities in PC and OFC change during odor habituation, but modulating neuronal activities in PC can also modify odor habituation behavior^10,12,14,15^. This suggests that the olfactory cortex is involved in odor habituation. Within 200 ms, the PC and OFC are activated mainly by odors^16,17^, and odor-specific information may be processed in the PC^28^. If the olfactory cortex processes odor signals within 200 ms and is involved in odor habituation, brain activity may change within 200 ms during odor habituation. Our data show that during odor habituation, the olfactory ERP signal within 200 ms changes, suggesting that during odor habituation, the olfactory cortex may modulate odor processing at very early time.

We also found that the odor signal during odor habituation is asymmetrically processed in the brain. In our study, the PP amplitude within 200 ms showed negative correlation with behavior mainly in the right hemisphere (Fig. 5a). These data are in line with the pattern of EEG topographical data at 155 ms in a previous study^16^, which reported activation of the secondary olfactory cortex, such as the inferior frontal OFC, left superior OFC, and left gyrus rectus. The asymmetric pattern of topographical data might also be supported by a previous fMRI study on odor habituation in human subjects, which found that the decrease in the activity of the PC was not the same bilaterally^15^. Interestingly, the positive correlation of the NP amplitude and the negative correlation of PP amplitude with the behavior data in the right hemisphere were maintained in our ERP data from (200 to 800) ms (Figs. S2a and S3a of the SI).

Patterns of NP and PP amplitudes between (200 and 800) ms were similar to those in previous odor habituation studies^22–25^. In these studies, which measured ERP in humans, the amplitudes of NP and PP of odors at (200–800) ms decreased during odor habituation. In our study, these amplitudes showed positive correlations with odor habituation, although the differences in the amplitude across the conditions in most of the channels were not significant (Figs. S2b and c and S3b of the SI). We also found that during odor habituation, the NP and PP amplitudes decreased in several channels within 200 ms (Table 2).

However, some results differed between our study and previous odor habituation ERP studies^22–25^. Although under habituation condition, NP and PP amplitudes of odors were not increased in those studies, our data showed that under our habituation condition (i.e. “Same”), the NP and PP amplitudes of odors were increased in some channels, and these patterns contributed to the negative correlation between ERP and odor habituation. Furthermore, in our data, positive and negative correlation between ERP and odor habituation were distributed, depending on the hemisphere, not only within 200 ms, but also at (200–800) ms (Figs. 4, 5, and Figs. S2 and S3 of the SI). The negative correlation between ERP and odor habituation, and asymmetrical topographical pattern in our data may be partially explained by previous odor habituation studies^15,16^, as mentioned above. However, our data was still different from those previous odor habituation ERP studies^22–25^. In addition, we found that during odor habituation, the latencies of NP and PP differed significantly. Those previous studies did not show such differences in ERP latency. One of the possible reasons is the respiration control method. In those previous studies, participants used a breathing technique called ‘velopharyngeal closure’, and received odors directly from tubes in the nose. However, in our study, we let participants breathe freely. Previous studies suggested that respiration may affect the speed of odor signal processing in the brain^33–35^. Another possible reason is that our experimental settings affected the data. In particular, we offered odors to participants in turn, and this setting might implicate the cognitive process of mismatch or oddball^36,37^. We offered odors twice in one trial (Fig. 1). Depending on the condition, the odors could be matched or mismatched between the first and second time. When a new or different stimulus was offered, it was processed faster^38^. However, neither of these reasons can explain the difference in the ERP latency between our study and previous studies.

Our ERP data showed smaller SNR than in previous olfactory ERP studies^23,39^. Although this could be an issue, in that ERP interpretation should be based on the actual signals, but not on the noise signals, our results were based on the ERP data that were statistically higher signals, compared to the noise level (Table 1). This SRN result suggests that our results were based on actual signals, rather than noise signals. One possibility of the smaller SNR of our data might be from the (40–200) ms period of our ERP data. Within 200 ms, the primary olfactory cortex and OFC are mainly activated by odors^16,17^. These brain areas are located at deep structures of the brain (Brodmann area 11, 27), and activities of these areas might be the main sources of our ERP signals.

The following points should be considered in interpreting our results related to odor habituation. Habituation studies in other sensory systems have suggested that during habituation, attention could affect the ERP signal and behavior^40,41^. Therefore, attention could have affected our data as well. To induce odor habituation, we offered odors to participants for 30 s. Participants may have paid less attention to previously perceived odors (“Same” condition), than to new or different odors (“None” and “Different” conditions). One of the other points is in regard to the “Different” condition. There were several channels for which the ERP amplitude or latency under “Different” condition were different from those under “None” condition. This difference in ERP data might imply the possiblity that cross adaptation between two odors could contribute to this difference, although the behavioral result showed no significant difference in perceived intensity between the “None” and “Different” conditions. Another point to consider is that there might be a difference in the odor signal in the peripheral system between our study and previous studies. We set the habituation step at 30 s, instead of changing the interval between offering the odors. If we used the olfactometer for offering the odors and measuring EEG data both in the habituation step and the test step, we might examine when the ERP of odors started to be changed during the odor habituation in our data. However, this is beyond the purposes of our study. This might more decrease the activities of the OSNs in the olfactory epithelium because of adaptation. Therefore, further studies are needed to solve these questions.

In conclusion, unlike previous studies, our study was focused mainly on ERP changes within 200 ms during odor habituation. We found that the NP and PP within 200 ms changed in relation to odor habituation. This result suggests clues to the role of the olfactory cortex in odor habituation within 200 ms, because during odor habituation, changes in ERP within 200 ms could represent changes in the perception of odor intensity. Moreover, odor habituation is processed asymmetrically in the brain. Because of the paucity of studies on how during habituation, odor signal is processed in the brain within 200 ms, our results could be meaningful in the understanding of early olfactory signal processing in the brain.

## Supplementary information

Supplementary Information.

## Data Availability

The datasets generated during and/or analysed during the current study are available from the corresponding author on reasonable request.
