# Supplementary material for: Odor habituation can modulate very early olfactory event-related potential
Source: Sci Rep. 2020 Oct 22;10:18117. doi: 10.1038/s41598-020-75263-7 (PMC7582193; doi:10.1038/s41598-020-75263-7)
Supplement: Supplementary file 1 — Supplementary Information. [file 41598_2020_75263_MOESM1_ESM.docx]

**Odor habituation can modulate very early olfactory event-related potential**

Kwangsu Kim^1,†^, Jisub Bae^1,†^, Youngsun Jin^2^ and Cheil Moon^1,3,*^

^1^Department of Brain and Cognitive Sciences, Graduate School, Daegu Gyeongbuk Institute of Science and Technology (DGIST), Daegu, Korea

^2^Department of Psychology College of Social Sciences Kyungpook National University, Daegu, Korea

^3^Convergence Research Advanced Centre for Olfaction, Daegu Gyeongbuk Institute of Science and Technology (DGIST), Daegu, Korea

† Both authors contributed equally to this work

* Corresponding author

Correspondence:

Cheil Moon, Ph.D., Department of Brain & Cognitive Sciences, Graduate School, Daegu Gyeungbuk Institute of Science and Technology, 333, Techno Jung-Ang Daero, Hyeonpung-Myeon, Dalseong-Gun, Daegu, 711-873, Korea. E-mail: cmoon@dgist.ac.kr; Tel: +82-53-785-6100; Fax: +82-53-785-6109

**Supplementary Information (SI)**

| **(a)** |  |  | | | |  | | | |
| --- | --- | --- | --- | --- | --- | --- | --- | --- | --- |
| **ERP component** | **Channel** | **Amplitude (µV)** | | | | **Latency (ms)** | | | |
|  |  | None | Different | Same | F-value | None | Different | Same | F-value |
| **N1**  NP at  (200–700) ms | **Right hemisphere** |  |  |  |  |  |  |  |  |
|  | C2 | –2.04 | –1.94 | –1.89 | 0.11 (ns) | 575 | 485 | 424 | 5.45* |
|  | **Left hemisphere** |  |  |  |  |  |  |  |  |
|  | CP1 | –2.42 | –1.90 | –2.11 | 4.070* | 439 | 474 | 490 | 0.29 (ns) |
| **(b)** |  |  |  |  |  |  |  |  |  |
| **P2**  PP at  (300–800) ms | **Right hemisphere** |  |  |  |  |  |  |  |  |
|  | FT8 | 2.51 | 3.20 | 3.85 | 4.02* | 563 | 532 | 546 | 0.12 (ns) |
|  | TP8 | 2.80 | 2.93 | 2.54 | 0.50 (ns) | 633 | 489 | 531 | 3.44* |
|  | **Left hemisphere** |  |  |  |  |  |  |  |  |
|  | PO7 | 4.97 | 4.00 | 3.97 | 1.39 (ns) | 653 | 534 | 593 | 3.45* |
|  | **Central position** |  |  |  |  |  |  |  |  |
|  | Pz | 2.19 | 3.07 | 2.62 | 5.58* | 498 | 552 | 572 | 0.97 (ns) |

**Supplementary Table S1. Channels with significant differences among the conditions in the amplitude and latency of N1 and P2.** In toto, 64 channels were used. **a.** Amplitude and latency of N1. Two channels showed significant differences in NP amplitude (CP1) or latency (C2). **b.** Amplitude and latency of P2. Four channels showed significant differences in P2 amplitude (FT8 and Pz) or latency (TP8 and PO7).


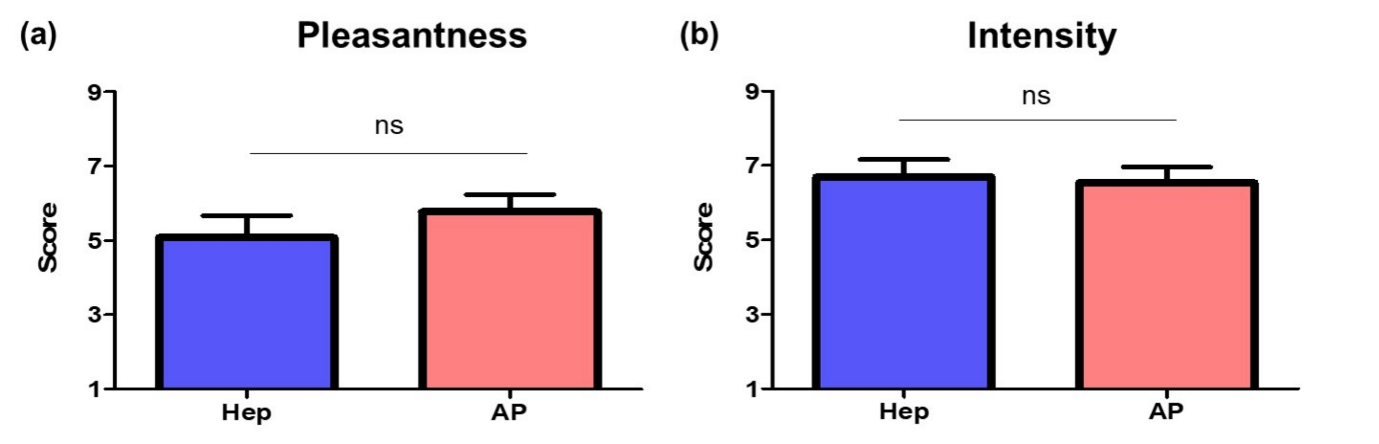


Supplementary Figure S1. No difference in intensity and pleasantness of two odors. Y-axes show the pleasantness and intensity scores of the two odors evaluated by using a 9-point Likert scale questionnaire. The pleasantness and intensity scores of heptanol (Hep) were not significantly different from those of 2-acetyl pyrazine (AP).


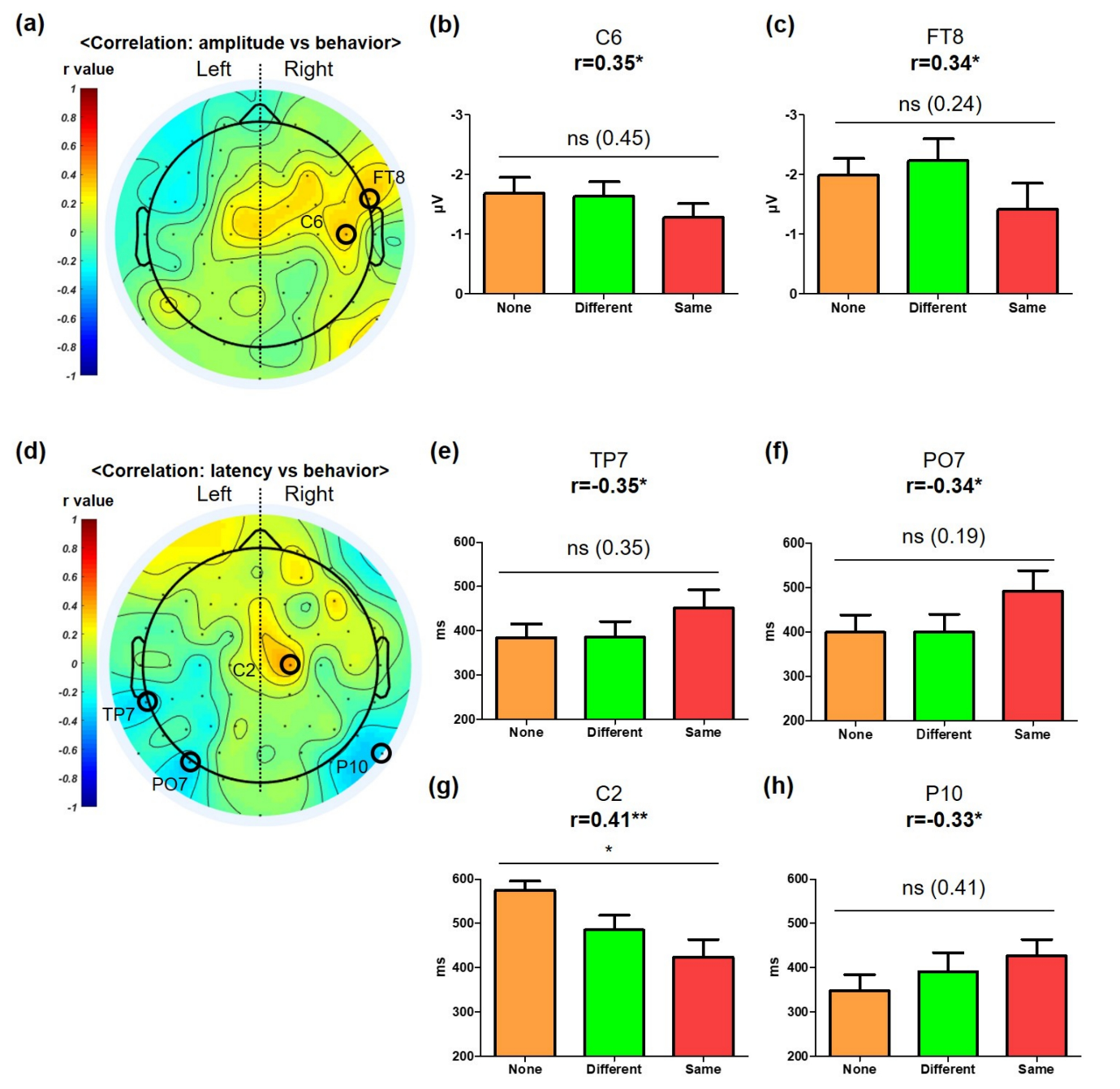

Supplementary Figure S2. Correlation pattern between N1 and behavior ((200–700) ms NP vs. behavior results). a. Topographical patterns of correlation between N1 amplitude and behavior. Two channels (encircled) showed significant correlations (C6 and FT8). b and c. C6 and FT8 significantly correlated with behavior (r-value ≥ 0.34). d. Topographical patterns of correlation between N1 latency and behavior. Encircled channels (TP7, PO7, C2, and P10) showed significant correlations. e–h. TP7, PO7, C2, and P10 significantly correlated with behavior (r-value ≥ 0.33), either positively (TP7, PO7, and P10), or negatively (C2). C2 also showed statistically significant differences across the conditions (latency in the “same” condition was the fastest). *p < 0.05, **p < 0.01.


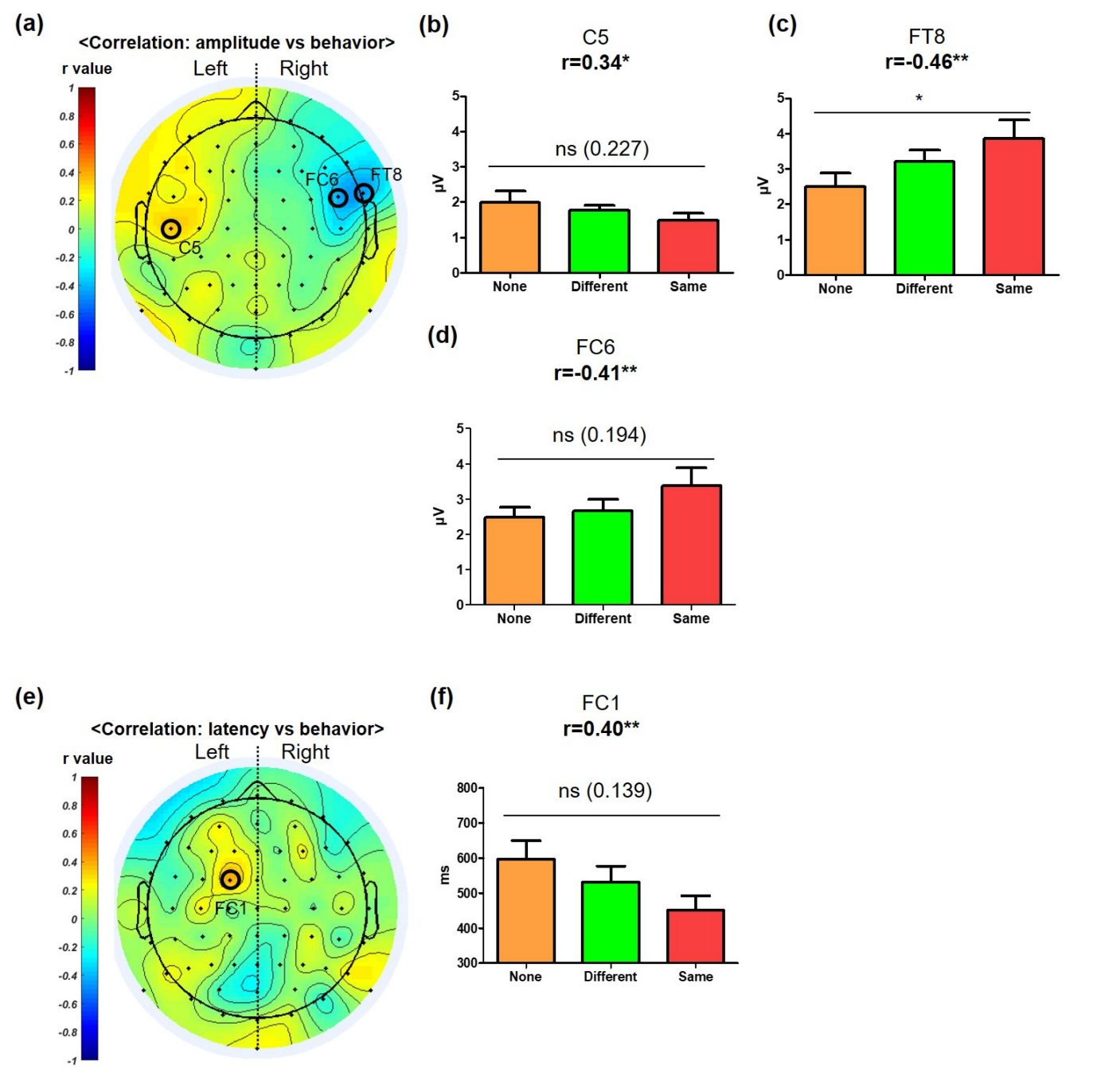


Supplementary Figure S3. Correlation pattern between P2 and behavior ((300–800) ms PP vs. behavior results). a. Topographical patterns of correlation between P2 amplitude and behavior. Three channels (encircled) showed significant correlations (C5, FT8, and FC6). b–d. C5, FT8, and FC6 significantly correlated with behavior (r-value≥ 0.34). FT8 also showed significant differences across the conditions. e. Topographical patterns of correlation between P2 latency and behavior. One channel (FC1) was significantly correlated with behavior. f. FC1 was significantly correlated with behavior (r value= 0.40), with no significant differences across the conditions. *p < 0.05, **p < 0.01.


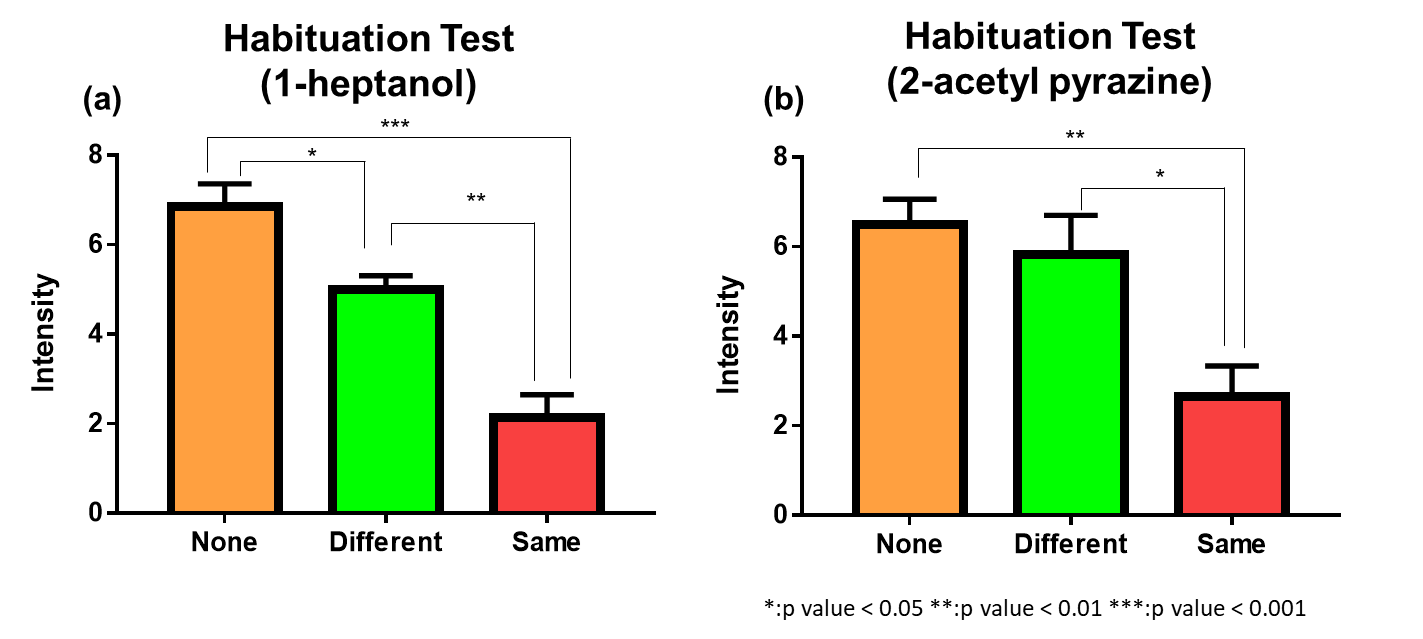


**Supplementary Figure S4. Decrease in odor intensity of each odorant when the same odorants were offered. a.** The intensities of 1-heptanol offered in the test step were compared across the three conditions. **b.** The intensities of 2-acetyl pyrazine offered in the test step were compared across the three conditions. *p < 0.05, **p < 0.01, ***p<0.001.


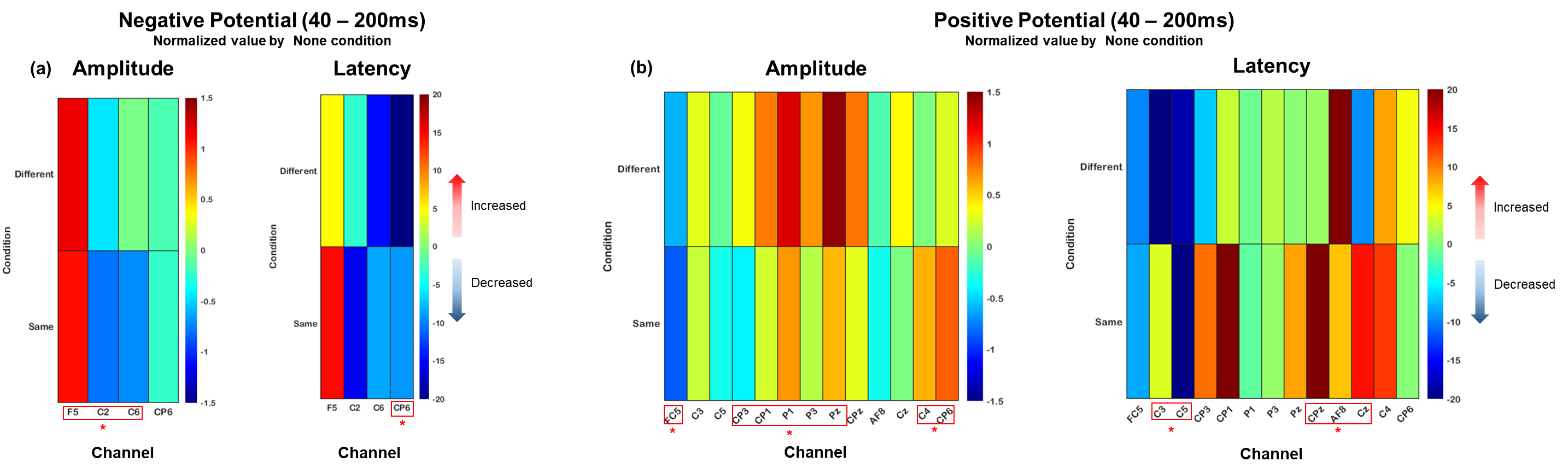


**Supplementary Figure S5. Heatmaps of channels with significant differences across the conditions in the amplitude and latency of NP and PP for (40–200) ms. a.** NP. Four channels showed significant differences in NP amplitudes or latency. **b.** PP. Thirteen channels showed significant differences in PP amplitudes or latency. “None” condition was subtracted as a baseline. *p < 0.05.
